# Electroencephalogram Access for Emotion Recognition Based on a Deep Hybrid Network

**DOI:** 10.3389/fnhum.2020.589001

**Published:** 2020-12-16

**Authors:** Qinghua Zhong, Yongsheng Zhu, Dongli Cai, Luwei Xiao, Han Zhang

**Affiliations:** ^1^School of Physics and Telecommunication Engineering, South China Normal University, Guangzhou, China; ^2^South China Academy of Advanced Optoelectronics, South China Normal University, Guangzhou, China

**Keywords:** electroencephalogram, access, emotion recognition, convolutional neural network, hidden markov model, deep hybrid network

## Abstract

In the human-computer interaction (HCI), electroencephalogram (EEG) access for automatic emotion recognition is an effective way for robot brains to perceive human behavior. In order to improve the accuracy of the emotion recognition, a method of EEG access for emotion recognition based on a deep hybrid network was proposed in this paper. Firstly, the collected EEG was decomposed into four frequency band signals, and the multiscale sample entropy (MSE) features of each frequency band were extracted. Secondly, the constructed 3D MSE feature matrices were fed into a deep hybrid network for autonomous learning. The deep hybrid network was composed of a continuous convolutional neural network (CNN) and hidden Markov models (HMMs). Lastly, HMMs trained with multiple observation sequences were used to replace the artificial neural network classifier in the CNN, and the emotion recognition task was completed by HMM classifiers. The proposed method was applied to the DEAP dataset for emotion recognition experiments, and the average accuracy could achieve 79.77% on arousal, 83.09% on valence, and 81.83% on dominance. Compared with the latest related methods, the accuracy was improved by 0.99% on valence and 14.58% on dominance, which verified the effectiveness of the proposed method.

## Introduction

In order to improve the reliability of HCI, researchers have always advocated for adding emotion-related components to the information processing network of robot brains (Pessoa, 2019; Xiao et al., 2020). With the development of HCI technology and cognitive neuroscience, the ability of robot brains to perceive human behavior is enhanced using these modern achievements in a brain-computer interface system (Korovesis et al., 2019). Therefore, it is of great significance to study EEG access for emotion recognition and its application in robot brains.

At present, the process of emotion recognition based on EEG access can be divided into the following steps, namely, induction of emotional states, acquisition and preprocessing of EEG signals, extraction and processing of EEG features, and emotion pattern learning and recognition (Koelstra et al., 2012). In general, the preprocessing of EEG signals involves the frequency and brain location of the selected signals. Fast Fourier transform (FFT) is a common frequency analysis method for EEG signals (Yin et al., 2017; Kwon et al., 2018). However, FFT cannot reflect temporal information in frequency data. Therefore, short-time Fourier transform (STFT), which could extract time-frequency domain features, is now used as an EEG emotion feature for emotion recognition (Liu et al., 2017). For example, wavelet transform, a typical STFT analysis method, is used to decompose and reconstruct EEG signals. The obtained wavelet energy is used as a feature for emotion recognition (Li et al., 2016). However, the human brain is a nonlinear dynamic system, and the EEG signals are difficult to analyze when using traditional time-frequency feature extraction and analysis methods. So, the asymmetry features regarding brain regions, such as DASM (differential asymmetry) and RASM (rational asymmetry) were explored for emotion recognition (Zheng et al., 2017). However, these methods only studied the relationship of symmetrical electrodes in the brain, and did not connect all the electrodes. In addition, the EEG signals were composed of rhythmic signals from different regions of the brain, which could reflect brain activity (Whitten et al., 2011). Hence, an EEG signal, which was decomposed into different frequency band signals, could be used for emotion recognition by the K nearest neighbor algorithm (KNN) (Li et al., 2018), support vector machine (SVM) (Zhuang et al., 2017), and an artificial neural network (ANN) (Mert and Akan, 2016). However, traditional machine learning algorithms cannot obtain the high-level abstract features of an EEG. In recent years, deep learning network methods have been applied to the EEG for emotion recognition. In terms of static models, depth features extracted from the CNN and statistical features selected by Pearson's correlation techniques were used for emotion recognition (Lee et al., 2020), which achieved an average accuracy rate of 80.90% on arousal and 82.10% on valence. The time-frequency feature map of each EEG channel was inputted into a 2D-CNN (Kwon et al., 2018), which achieved an average accuracy rate of 78.12% on arousal and 81.25% on valence. The frequency domain, spatial, and frequency band features of EEG signals were fed into the capsule network (CapsNet) (Chao et al., 2019), which achieved an average accuracy rate of 68.28% on arousal, 66.73% on valence, and 67.25% on dominance. However, these static models cannot extract the temporal information of EEG features effectively. As for dynamic time models, a HMM model was used to establish the relationship between current and previous emotional states (Chen et al., 2015), which achieved an average accuracy rate of 73.00% on arousal and 75.63% on valence. Then, a hybrid neural network model was created, composed of a CNN and a recurrent neural network (Li et al., 2016), which achieved an average accuracy rate of 74.12% on arousal and 72.06% on valence. And a recurrent neural network for long short-term memory (LSTM-RNN) was used for emotion recognition (Xing et al., 2019), which achieved an average accuracy rate of 81.10% on arousal and 74.38% on valence. But these dynamic models cannot effectively extract the spatial information of EEG features, which have a low performance for emotion recognition based on EEG access.

Therefore, a method based on MSE and deep hybrid network CNN-HMMs was proposed in this paper for EEG emotion recognition. By taking the advantages of a HMM model on tracking time series signals, high-level features from the CNN could be modeled and classified by HMMs. In addition, according to the position of brain electrodes, multi-band spatial feature matrices were constructed and fed into the deep hybrid network CNN-HMMs for emotion recognition.

## Principle

### EEG Feature Extraction

#### Frequency Pattern Decomposition

EEG signals are composed of brain rhythm signals, event-related potentials (ERP), and spontaneous electrical activity signals, and changes of brain states are often characterized by rhythmic signals from different brain regions (Whitten et al., 2011; Koelstra et al., 2012; Wang et al., 2014). The EEG signal can be decomposed into four frequency band signals by Butterworth filters, which are the θ wave (4–7 Hz), α wave (8–13 Hz), β wave (14–30 Hz), and γ wave (31–45 Hz). The properties of the Butterworth filter include a maximally flat magnitude response in the passband region, and a gain of 0 dB at direct current (DC). The magnitude-squared response |*H*(*w*)|^2^, which is an integer order Butterworth filter of order *n*, is given by Equation (1) (Mahata et al., 2018).

(1) $$\begin{array}{l} {\left|H\left(w\right)\right|}^{2}=\frac{1}{1+{\left(w/w_{c}\right)}^{2n}}=\frac{1}{1+\varepsilon^{2}{\left(w/w_{p}\right)}^{2n}} \end{array}$$

Where *n* is the order of the filter, *w* is the digital domain frequency, *w*_*c*_ is the cut-off frequency, *w*_*p*_ is the passband edge frequency, and ε is the ripple parameter.

#### MSE Algorithm

MSE analysis was used to estimate the complexity of irregular physiological time series at different time scales (Costa et al., 2002, 2005). The calculation processes of the MSE are shown as follows.

The EEG sequences are transformed to different time scales by different scale factors. For the given length of EEG sequence ***X*** = {*x*_1_*, x*_2_*,…, x*_*N*_}, the EEG sequence ***Y*** with scale factor τ is obtained by scale transformation. The scale transformation process is shown in Equation (2).

(2) $$\begin{array}{l} y_{j}^{\tau }=\frac{1}{\tau }\overset{j+\tau -1}{\underset{i=j}{\sum }}x_{i},\;1\leq j\leq N-\tau +1;\;1\leq i\leq N;\tau \in N^{+} \end{array}$$

Where *N* is the length of the sequence and τ is the scale factor. When τ = 1, the resulting sequence is the raw EEG sequence ***X***. When τ > 1, the raw EEG sequence can be converted into the sequence $Y=\left\{y_{1}^{\tau },y_{2}^{\tau },\ldots ,y_{N-\tau +1}^{\tau }\right\}$, its length is no more than *N*–τ+1.

For the EEG sequence ***Y*** at the scale of τ, the absolute value of the maximum difference $d\left[Y_{i}^{\tau },\;Y_{j}^{\tau }\right]$ between the elements of vector $Y_{i}^{\tau }$ and vector $Y_{j}^{\tau }$ is shown in Equation (3).

(3) $$\begin{array}{l} d\left[Y_{i}^{\tau },\;Y_{j}^{\tau }\right]=\underset{k=0}{\overset{m-1}{\max}}\left(\left|y_{i+k}^{\tau }-y_{j+k}^{\tau }\right|\right),\;1\leq i,\;j\leq N-m+1 \end{array}$$

where $Y_{i}^{\tau }=\left\{y_{i+1}^{\tau },y_{i+2}^{\tau },\ldots ,y_{i+m-1}^{\tau }\right\}$ is a set of *m* dimension vectors, $y_{i+k}^{\tau }$ is the element of the vector $Y_{i}^{\tau }$, and $y_{j+k}^{\tau }$ is the element of the vector $Y_{j}^{\tau }$, but$Y_{i}^{\tau }\neq Y_{j}^{\tau }$. For the given similarity tolerance *r*(*r* > 0), the similarity $B_{i}^{m}\left(r,\;\tau \right)$ between the vector $Y_{i}^{\tau }$ and the vector $Y_{j}^{\tau }$ is shown in Equation (4).

(4) $$\begin{array}{l} B_{i}^{m}\left(r,\;\tau \right)=\frac{B_{i}^{\tau }\left(r,\;\tau \right)}{N-m}=\frac{num\left\{d\left[Y_{i}^{\tau },\;Y_{j}^{\tau }\right]<r\right\}}{N-m} \end{array}$$

where $B_{i}^{\tau }\left(r,\;\tau \right)$ is the number of $num\;\left\{d\left[Y_{i}^{\tau },\;Y_{j}^{\tau }\right]<r\right\}$. Then, the average similarity $B_{i}^{m}\left(r,\;\tau \right)$ can be calculated by Equation (5) at the scale of τ.

(5) $$\begin{array}{l} B^{m}\left(r,\;\tau \right)={\left(N-m+1\right)}^{-1}\overset{N-m+1}{\underset{i=1}{\sum }}B_{i}^{m}\left(r,\;\tau \right) \end{array}$$

In Equations (3–5), the dimension *m* is changed to *m* + 1. The average similarity *B*^*m*+1^(*r*, τ) can be calculated by the equations of (3), (4), and (5). Then, the MSE value of the raw EEG sequence ***X*** can be calculated as Equation (6).

(6) $$\begin{array}{l} MSE=-\text{ln}\left(B^{m+1}\left(r,\;\tau \right)/B^{m}\left(r,\;\tau \right)\right) \end{array}$$

where the settings of parameters *m* = 2 and *r* = 0.2 × *std* (*std* is a standard deviation of the time series) are the best choice in analyzing the EEG signals (Richman and Moorman, 2000). Thus, the settings of the *m* = 2 and *r* = 0.2 × *std* are used in this paper.

### Deep Hybrid Network CNN-HMMs

**Figure 1:**
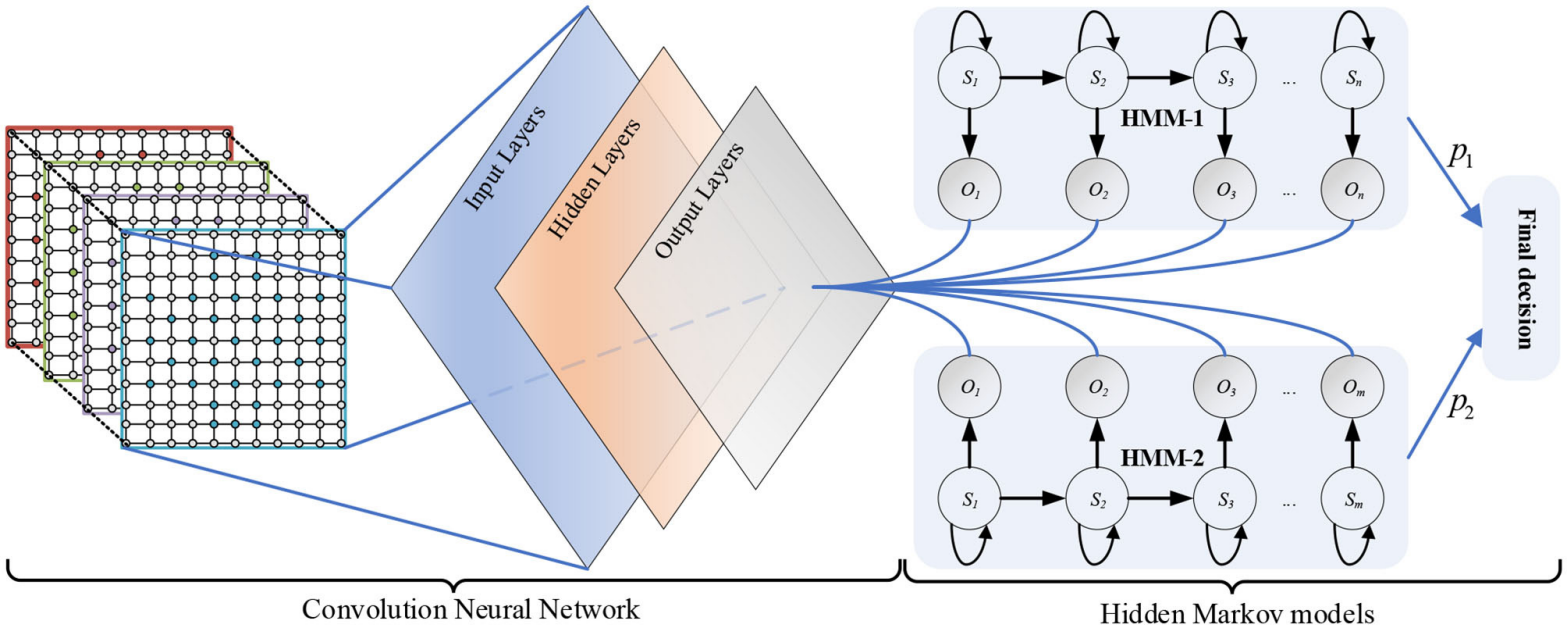
Schematic diagram of the deep hybrid network CNN-HMMs.

The deep hybrid network CNN-HMMs is composed of a CNN and two HMMs. As shown in Figure 1, the CNN contains input layers, hidden layers, and output layers. In addition, sequence *S*_1_ = *s*_1_, *s*_2_, …, *s*_*n*_ is the implicit state of HMM-1, and sequence *O*_1_ = *o*_1_, *o*_2_, …, *o*_*m*_ is the observable state of HMM-1. Sequence *S*_2_ = *s*_1_, *s*_2_, …, *s*_*m*_ is the implicit states of HMM-2, and sequence *O*_2_ = *o*_1_, *o*_2_, …, *o*_*m*_ is the observable state of HMM-2.

#### Structure of CNN

**Figure 2:**
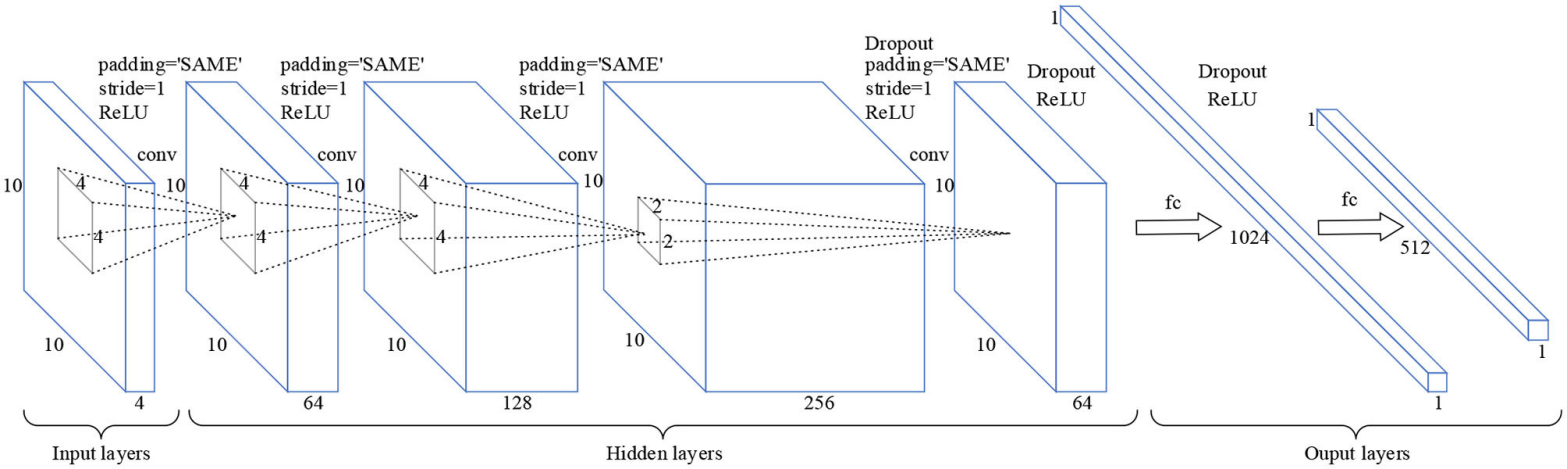
Schematic diagram of the CNN model. *Padding* = “*SAME”* means that zero padding is used to prevent information from getting lost at edges of the cube. *Stride* = *1* means that the step size of each convolution operation is 1. *Dropout* means that hidden neurons are randomly deleted in the network.

The CNN is a kind of neural network which can be used to generate feature hierarchy, it has two significant characteristics: sparse connection and weight sharing (Lecun et al., 2015). The sparse connection can be used to extract the features of different regions in input layers, while weight sharing can greatly reduce the number of training parameters and training time, and simplify the network structure. As shown in Figure 2, the input layers are a 3D MSE matrix of a size 10 × 10 × 4, where 10 × 10 is the size of the single frequency band square matrix, and 4 is the number of the EEG frequency bands. In the hidden layers, the sizes of the four convolution layers are 10 × 10 × 64, 10 × 10 × 128, 10 × 10 × 256, and 10 × 10 × 64, respectively. And the convolution kernel sizes of each convolutional layer are 4 × 4, 4 × 4, 4 × 4, and 2 × 2, respectively. In the output layers, the sizes of the two connection layers are 1 × 1024 and 1 × 512, respectively. Moreover, the layer activation function is a rectified linear unit (*RELU*). So, the CNN has the ability of nonlinear feature transformation. And the function *RELU* is shown in Equation (7).

(7) $$\begin{array}{l} RELU\left(x\right)=max\left(x,\;0\right)=\left\{ \begin{array}{c} x,\;if\;x>0\\ 0,\;otherwise \end{array}\right. \end{array}$$

where the linear function *RELU*(*x*) is 0 when *x* < 0.

#### HMM Classifiers

**Figure 3:**
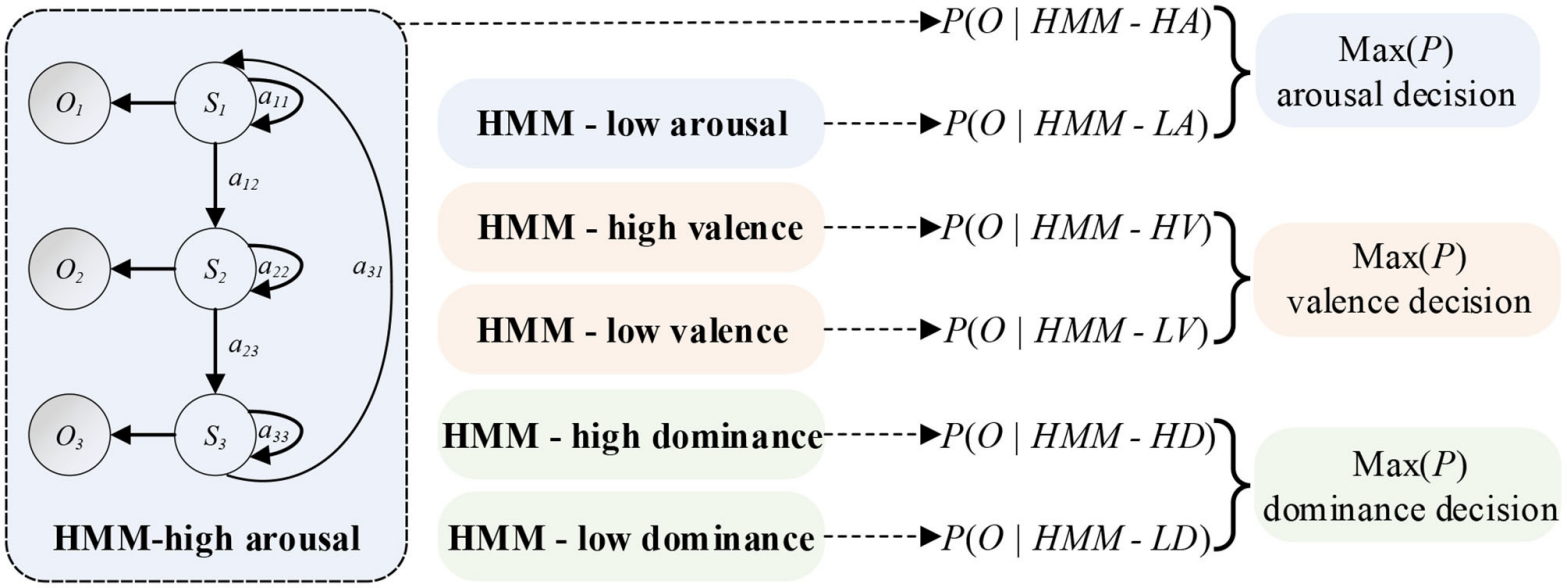
Schematic diagram of HMM classifiers. A HMM classifier can be created for an emotion state. The classifier is made up of *Q* hidden states which can generate observation variables *O*_*t*_ at each time point *t*. An observation sequence ***O*** can be obtained by regulating state transition probabilities *a*_*ij*_ and observation probability distributions. Final decision MAX(*P*) can be treated as the maximum probability of each emotional state.

A HMM has the ability of modeling time series. So, the EEG feature sequences can be treated as the Markov observation sequence *O* = *O*_1_, *O*_2_, …, *O*_*k*_, and the EEG emotional states can be treated as states *S* = *S*_1_, *S*_2_, …, *S*_*k*_ of a Markov process. λ = (π, ***A***, ***B***) can be defined as the HMM. Key parameters of the λ are the initial state probability distribution π = *p(q*_0_ = *S*_*i*_*)*, the transition probabilities *a*_*ij*_ = *p*(*q*_*t*_ = *S*_*j*_|*q*_*t*_1__ = *S*_*i*_) of the state transition matrix ***A***, and a model to estimate the observation probabilities *b*_*j*_(*k*) = *p*(*O*_*k*_|*S*_*j*_) of the observation probability matrix ***B***. The learning parameters of HMM can be realized using the Baum-Welch algorithm (Rabiner, 1990) based on the maximum likelihood estimation (MLE). Then, the objective function Equation (11) can be optimized by updating Equations (8–10). The parameters (π, ***A***, ***B***) can be obtained at the end. As shown in Figure 3, the output probability *P* of each HMM classifier can be obtained by Equation (11).

(8) $$\begin{array}{l} \bar{\pi_{i}}=\gamma_{1}\left(i\right),\;1\leq i\leq N \end{array}$$

where $\bar{\pi_{i}}$ is the initial state probability, and γ_1_(*i*) is the probability of state *S*_*i*_ at time *t* = 1.

(9) $$\begin{array}{l} \bar{a_{i,j}}=\frac{\overset{T-1}{\underset{t=1}{\sum }}\xi_{t}\left(i,\;j\right)}{\overset{T-1}{\underset{t=1}{\sum }}\gamma_{t}\left(i\right)},\;1\leq i,\;j\leq N;\;1\leq t\leq T-1 \end{array}$$

where $\bar{a_{i,j}}$ is the state transition probability, ξ_*t*_(*i, j*) is the state transition probability from state *S*_*i*_ at time *t* to state *S*_*j*_ at time *t* + 1, and γ_*t*_(*i*) is the probability of state *S*_*i*_ at time *t*.

(10) $$\begin{array}{l} \bar{b_{j}}\left(k\right)=\frac{\overset{T}{\underset{t=1,O_{t}=O_{k}}{\sum }}\gamma_{t}\left(j\right)}{\overset{T}{\underset{t=1}{\sum }}\gamma_{t}\left(j\right)},\;1\leq j\leq N;\;1\leq t\leq T \end{array}$$

Where $\bar{b_{j}}\left(k\right)$ is the observation probability of symbol *O*_*k*_ in state *S*_*i*_, and γ_*t*_(*j*) is the probability of state *S*_*j*_ at time *t*.

(11) $$\begin{array}{l} P\left(O|\lambda \right)=\underset{k=1}{\overset{T}{\prod }}p\left(O^{k}|\lambda \right)=\underset{k=1}{\overset{T}{\prod }}p_{k},\;1\leq k\leq T \end{array}$$

Where *P*(*O*|λ) is the maximum likelihood estimate probability, *O*^(*k*)^ is the symbol of sequence ***O***.

## Results and Discussion

In this part, we introduce the experimental processes and compare our method with other methods. Then, we evaluate the effectiveness of our framework on the DEAP dataset. Without loss of generality, the performance of emotional recognition based on EEG access was analyzed by a 10-fold cross-validation technology.

### Experimental Environment and Experimental Dataset

Table 1 shows the specific experimental environment for the experiments.

**Table 1:** Specific experimental environment.

**Name**	**Version**
CPU	Intel Core i7-9750H @2.60GHz
GPU	NVIDIA GeForce RTX 2060 6GB
RAM	DDR4 16GB
OS	Windows 10
Frameworks	Tensorflow-GPU 1.14.0, MATLAB 2019b

**Figure 4:**
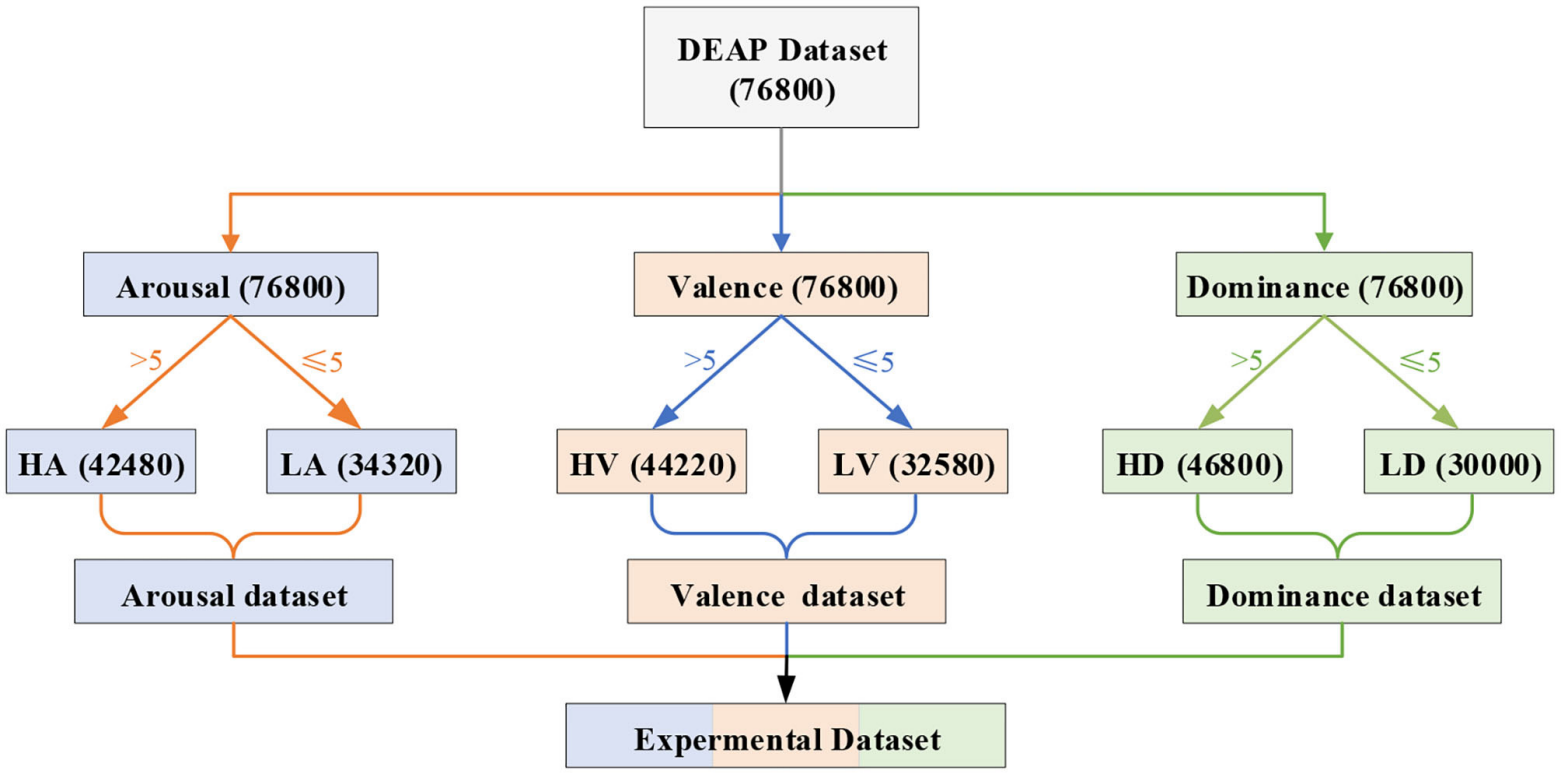
Schematic diagram of the experiment labels and data division. We obtained 76,800 (32 × 40 × 60) samples of each EEG band from the DEAP dataset. The number 32 represents 32 subjects, 40 represents 40 videos, and 60 represents that we divided the original 60 s of EEG signals into 60 equal segments. According to the emotional dimension level classification, there are 34,320 samples in LA, 42,480 samples in HA, 32,580 samples in LV, 44,220 samples in HV, 30,000 samples in LD, and 46,800 samples in HD.

The effectiveness of the proposed emotion recognition method was verified using the DEAP dataset (Koelstra et al., 2012). In the dataset, 63 s of EEG data were recorded for 32 subjects who watched 40 videos. The first 3 s of data were pre-trial baseline signals, and the last 60 s of data were trail signals. In addition, we classified the emotional states according to the scores of arousal, valence, and dominance. As shown in Figure 4, we divided the emotion recognition of EEG into three binary classifications. If the scores of arousal (or valence or dominance) were less than or equal to 5, the label was marked as low. If the scores were greater than 5, the label was marked as high. Thus, there were six labels on three emotional dimensions, namely, high arousal (HA), low arousal (LA), high valence (HV), low valence (LV), high dominance (HD), and low dominance (LD). We divided 60 s of EEG raw signals of a specific channel into 60 equal segments by 1 s sliding windows. Thus, all 60 divided segments of 1-second EEG signals had the same label as the original signals.

### Construction of a 3D MSE Feature Matrix

**Figure 5:**
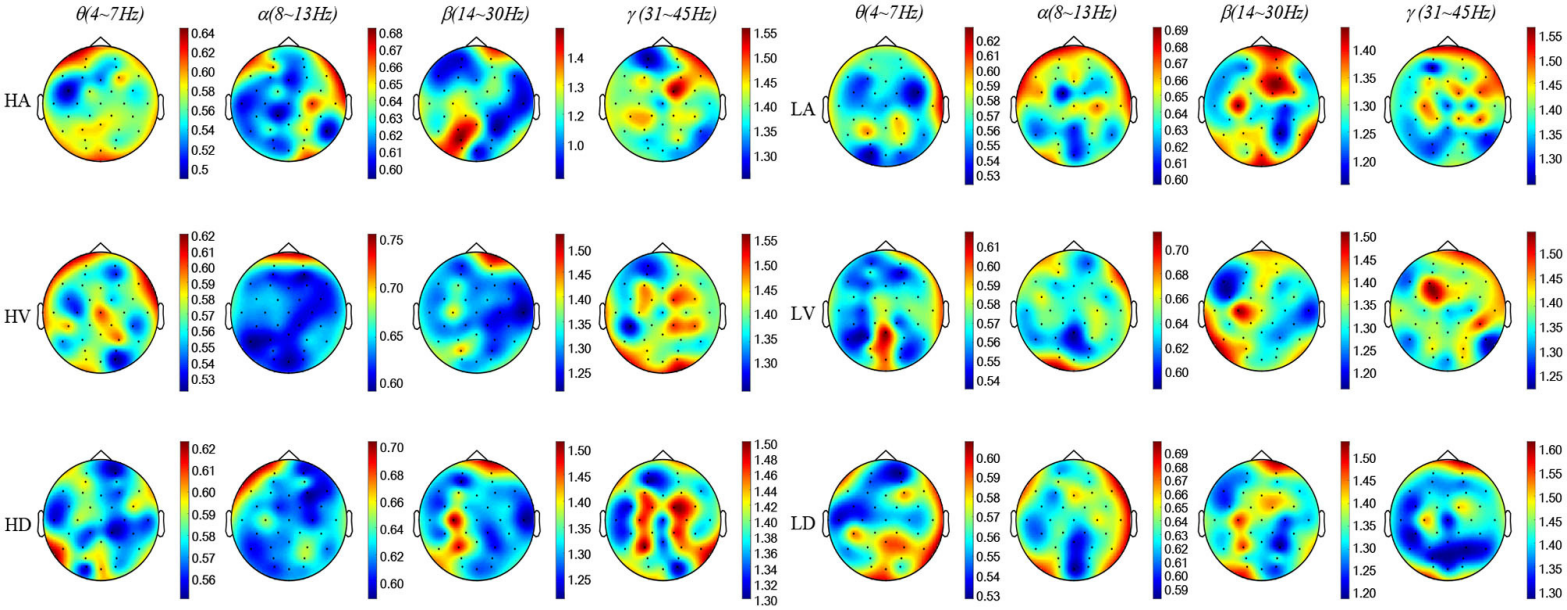
The MSE topographic map schematic of EEG samples. The color-bar represents the MSE value range of each topographic map. θ is 0.5 ~ 0.62, α is 0.60 ~ 0.70, β is 1.20 ~ 1.50, and γ is 1.20 ~ 1.60.

To present the distinctive MSE features, we used Pearson's correlation to calculate the correlation (*R*) between the low level and high level of each emotional state. When the time scale τ was 1, we calculated the average MSE values of the EEG samples with statistical significance *R* < 0.05 and drew the MSE map. As shown in Figure 5, the MSE value became larger with the increasing EEG frequency, which indicated that the complex components of the EEG signal were increasing. Thus, in order to extract more effective EEG emotional features, the MSE was used for emotional feature extraction in this paper.

**Figure 6:**
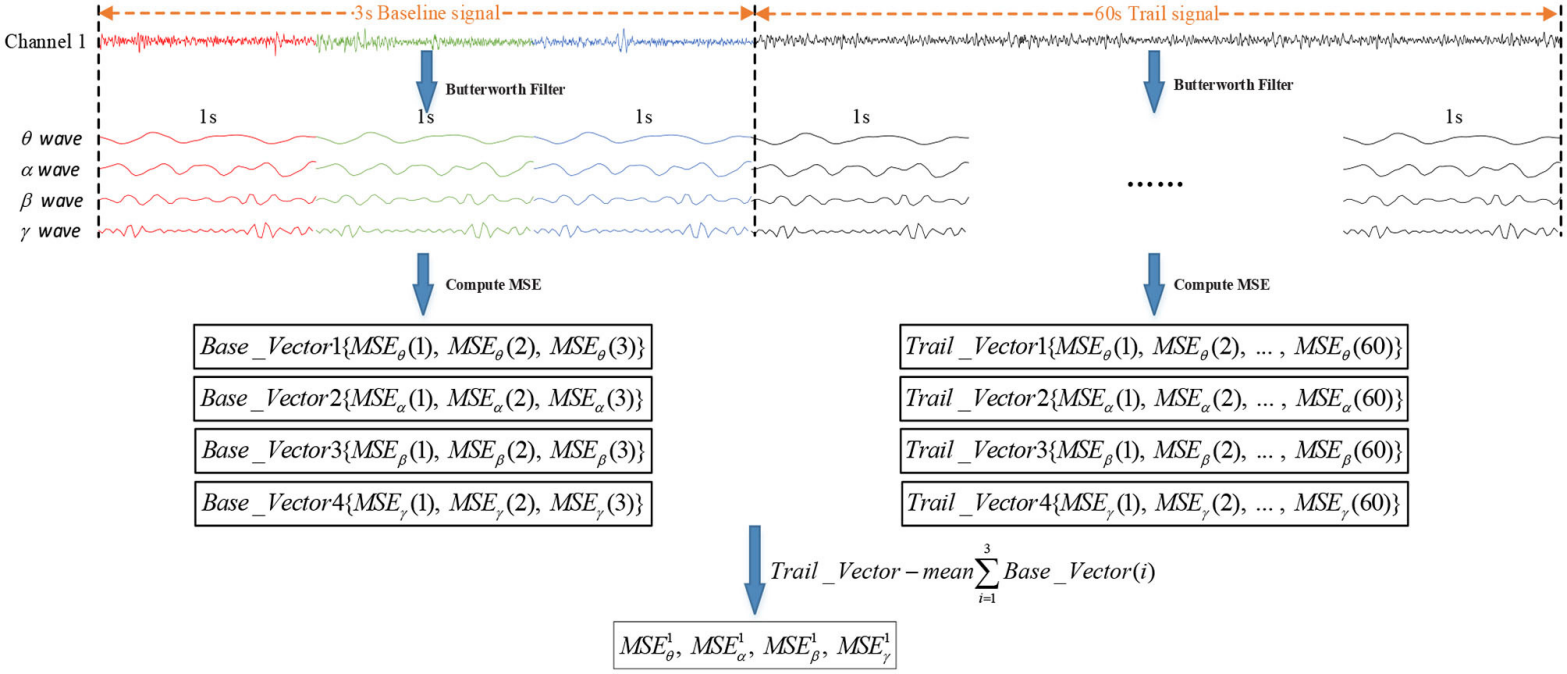
Process of removing the influence of baseline signals. *Base_Vector (i)* is the MSE value of baseline signals, *Mean*∑*Base*_*Vector* (*i*) is the MSE mean value of the 3-segment baseline signals, and *Trail_Vector (i)* is the MSE value of trail signals.

Before the MSE of the EEG signal was calculated, EEG signals were usually divided into short time frames within a window size of 1 second (Wang et al., 2014; Li et al., 2017). In order to improve the recognition accuracy, the divided trail signals needed to be removed from the baseline signals. As shown in Figure 6, every second of the raw EEG signals were decomposed into θ waves, α waves, β waves, and γ waves by Butterworth filters. Then, the MSE value of the 3-segment baseline signals and 60-segment trail signals were calculated by the MSE algorithm. Finally, the MSE could remove the influence of the baseline signals by calculating the difference between *Mean*∑*Base*_*Vector*(*i*) and *Trail_Vector (i)*.

**Figure 7:**
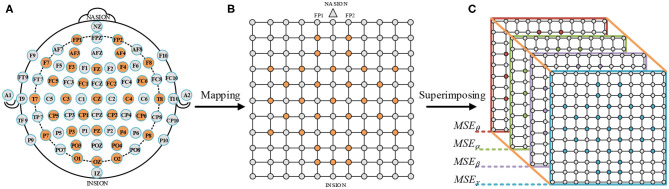
Construction process of a 3D MSE feature matrix. **(A)** International 10–20 system (Jasper, 1958). **(B)** A 2D square matrix of 32 EEG channels. **(C)** A 3D feature matrix of combining MSE_θ_, MSE_α_, MSE_β_, and MSE_γ_.

The MSE_θ_, MSE_α_, MSE_β_, and MSE_γ_ of the 32 EEG channels fill the orange positions in Figure 7B. In addition, the EEG electrodes circled in orange were testing points used in the DEAP dataset, as shown in Figure 7A. We connected the electrodes of the international 10-20 system (Jasper, 1958) with the testing electrode of the DEAP dataset. And then, a *N* × *N* square matrix was constructed (*N* is the maximum number of points between horizontal or vertical test points). Moreover, in order to avoid the loss of edge information, a layer of gray untested points was added to the outer layer of the matrix, as shown in Figure 7B. The gray points were filled with zero values. Next, we obtained four 2D square matrices (10 × 10). Finally, a 3D feature matrix of a size 10 × 10 × 4 was constructed by superimposing the four 10 × 10 square matrices of the EEG frequency bands in Figure 7C.

### Training CNN-HMMs and Parameters Selection

The recognition performance was analyzed using 10-fold cross validation technology. The obtained 76800 3D feature matrices were divided into ten equal groups. Nine groups were assigned to the training dataset, and the remaining one was assigned to the test dataset. All the feature matrices were fed into the deep hybrid network CNN-HMMs. The pseudo code of the detailed procedures for EEG emotion recognition are listed in Table 2. For the developed model with optimized parameters, the training time and the testing time were 134.33s and 35.45s, respectively.

**Table 2:** Pseudo code of the detailed procedures for EEG emotion recognition.

**Read** EEG Feature dataset and corresponding labels
**Start model structural identification**
Initialize CNN parameters and the learning rate
**def** conv_1, conv_2, conv_3, conv_4, cnn_fc1, cnn_fc2
**def** cnn_fc_drop, L2 regularization, cost_func, AdamOptimizer
**def** hmm_hight_model = hmm.GaussianHMM(components, iter, tol, covariance_type)
**def** hmm_low_model = hmm.GaussianHMM(components, iter, tol, covariance_type)
**End model structural identification**
**Start training CNN-HMM model**
**for** fold *=* 1: 10
**for** epoch = 1: training_epochs
**for** train_batch_num = 1: batch_num_per_epoch // CNN training
**Assign** cnn_batch, cnn_labels
session.run([cnn_fc2, cost], feed_dict={cnn_in: cnn_batch, cnn_labels})
**end for**
hmm batch = cnn_fc2;
**Assign** HMM train dataset, HMM test dataset
hmm_hight_model.fit(hmm_high_batch) // HMM training of hight-level emotion
hmm_low_model.fit(hmm_low_batch) // HMM training of low-level emotion
high_score = hmm_hight_model.score(test_dataset) // get test probability
low_score =hmm_low_model.score(test_dataset) // // get test probability
**compare** [high_score, low_score] **with** [high_labels, low_lables]
update learning rate
**end for**
**end for**
**End training CNN-HMM model**

For the CNN, the training process of the CNN consisted of optimizing parameters in the network. To prevent the CNN from over fitting in the learning process, a dropout technology and L2 regularization mechanism were introduced into a fully connected layer of the network. The value of *Dropout* was set to 0.5 and the learning rate was initialized to 0.01. When the verification errors of the network stopped dropping, the learning rate was divided by 10 until the iteration stopped.

For the HMM, we built two HMMs through the hmmlearn library of Python. A HMM classifier was created for an emotional state. The number of iterations was set to 1000. The stop threshold was set to 0.01. The learning parameters of HMM could be realized using the Baum-Welch algorithm. Parameters in HMMs were optimized in the training phase. Firstly, the transformation matrix ***A*** was represented as a Bakis model (Wissel et al., 2013) in which non-zero elements were only allowed in the upper triangle part. In this structure, the three transitions were looping (*a*_11_), jumping to the next state (*a*_12_), and skipping (*a*_31_), as shown in Figure 3. Then, we set experiments to explore the optimal Gaussian mixture component *M* and the feature dimension of each emotional state.

**Figure 8:**
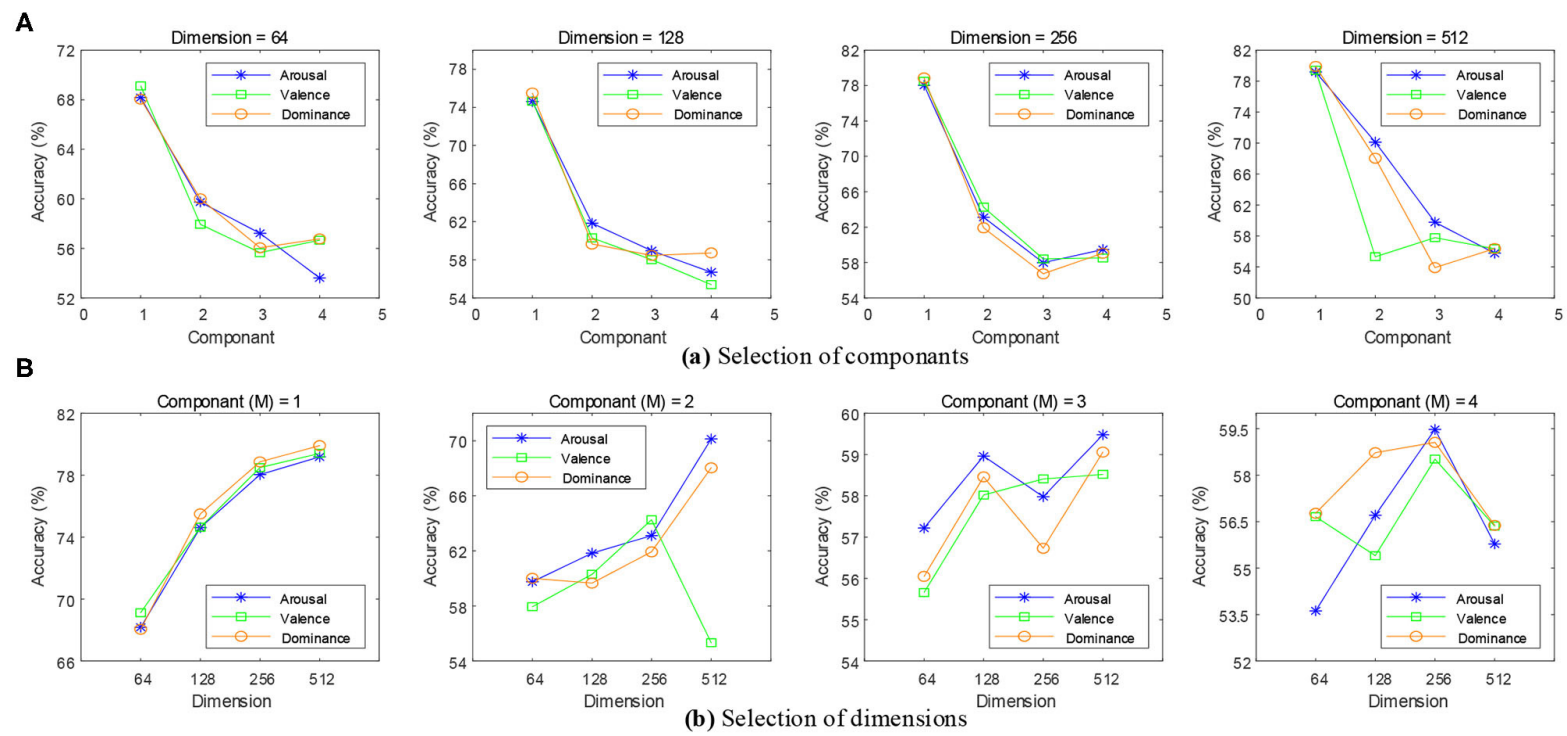
Selection of feature dimensions and Gaussian mixture components. **(A)** When the feature dimensions were 64, 128, 256, and 512, the emotion recognition accuracy of each mixture component can be obtained. **(B)** When the mixture components were 1, 2, 3, and 4, the emotion recognition accuracy of each feature dimension can be obtained.

When the time scale was τ = 1 for the MSE features, the Gaussian mixture component *M* was set to 1, 2, 3, and 4, and the feature dimension of the state Q was set to 64, 128, 256, and 512. As shown in Figure 8A, the accuracy of the emotion recognition showed a decreasing trend with the increase of *M*. The highest average recognition accuracy rate was obtained when *M* was 1. As shown in Figure 8B, the accuracy of emotion recognition was the highest with a steady upward trend, and the maximum accuracy rate was obtained when the feature dimension was 512. When *M* > *1*, the emotion recognition accuracy rate presented an unstable state. The result showed that the increase of *M* would reduce the quality of the model estimation, and a higher accuracy rate was obtained for a small number of states which contained only a few mixture components.

In order to obtain a robust generalization ability for the HMMs, the optimal feature dimension was 512 and *M* was 1. In addition, we also needed to set experiments to find the optimal time scale of the MSE.

### Time Scale τ Selection of MSE

**Figure 9:**
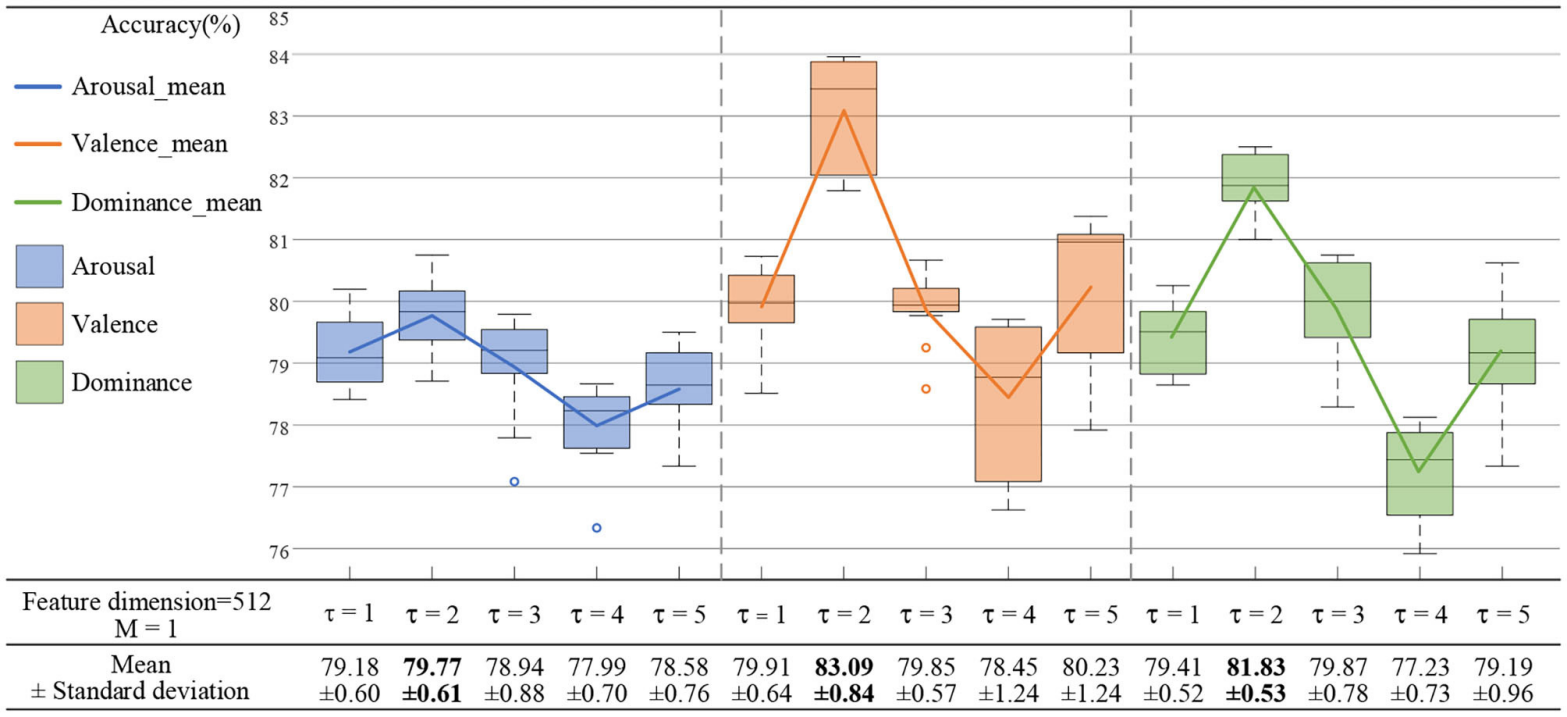
Average accuracy rate of emotion recognition at τ = 1 ~ 5.

In order to find the optimal performance of the deep hybrid network CNN-HMMs at the appropriate time scale, the MSE value was calculated by five time-scales, and the average accuracy rate of emotion recognition was obtained on arousal, valence, and dominance. As shown in Figure 9, the average accuracy rate increased at first, then decreased and then increased again. When τ was 2, the deep hybrid network CNN-HMMs could yield the highest average accuracy on arousal, valence, and dominance, which were 79.77, 83.09, and 81.83%. Therefore, the optimum time-scale of MSE was τ = 2.

### Results of EEG Access for Emotion Recognition

To verify the reasonableness of the proposed method, two groups of experiments were designed to perform emotion recognition on arousal, valence, and dominance.

**Figure 10:**
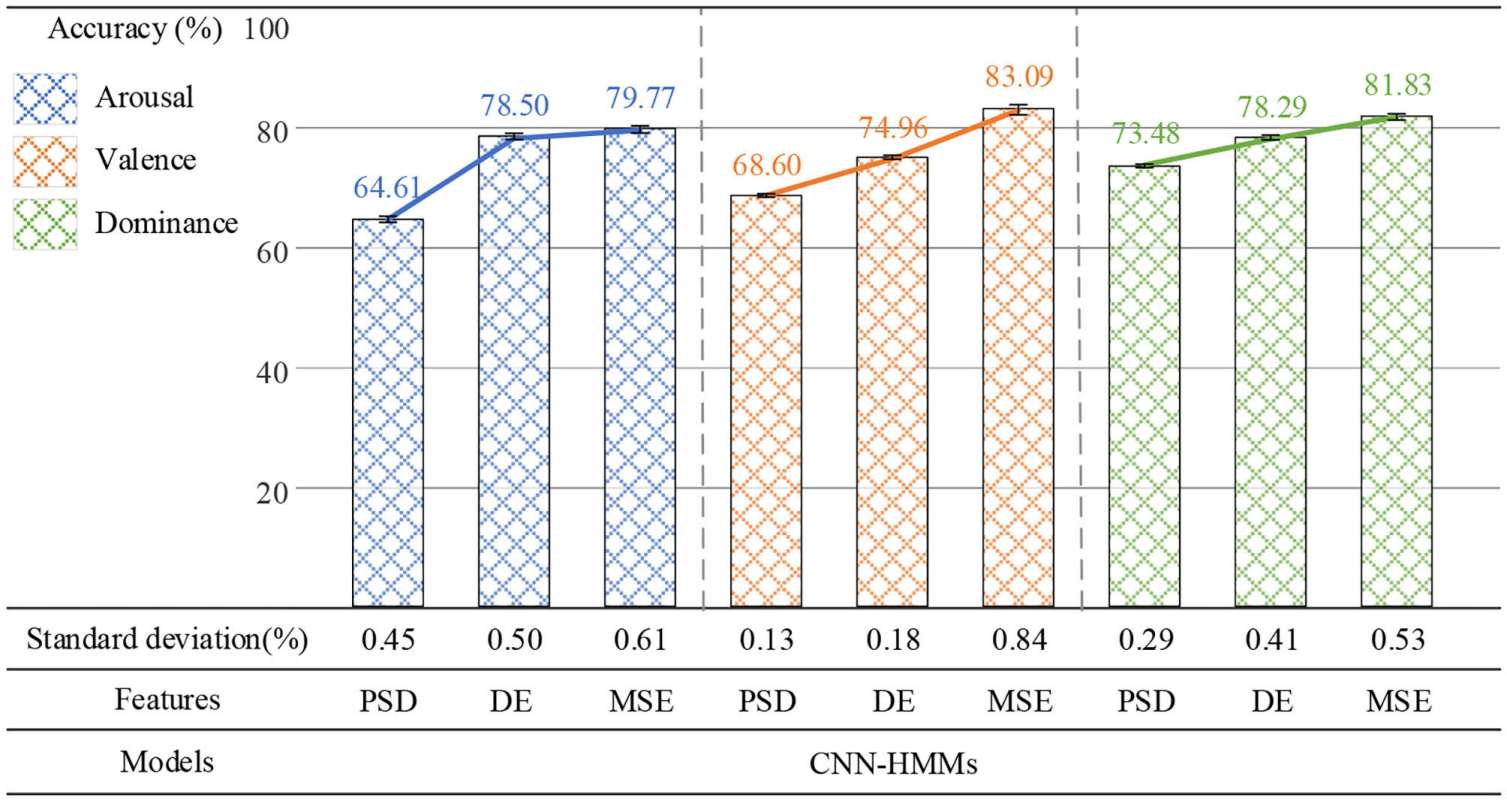
The results of the emotion recognition based on PSD, DE, and MSE.

In the first group of experiments, MSE, power spectral density (PSD), and differential entropy (DE) were used as EEG emotion features, and CNN-HMMs was used for recognition emotion. As shown in Figure 10, when PSD was used as the emotion feature of EEG, the average recognition accuracy rate of the deep hybrid network CNN-HMMs was 64.61% on arousal, 68.60% on valence, and 73.48% on dominance. When DE was used as the EEG emotion feature, 78.50, 74.96, and 78.29% were obtained. When MSE was used as the EEG emotion feature, optimal accuracy rates of 79.77, 83.09, and 81.83% were obtained. PSD was a time-frequency analysis method, while both DE and MSE were nonlinear dynamics analysis methods. In the proposed method, both MSE and DE were more effective in emotion recognition than PSD, which indicated that the emotional features based on EEG signals could be effectively extracted by the method of nonlinear dynamics.

**Figure 11:**
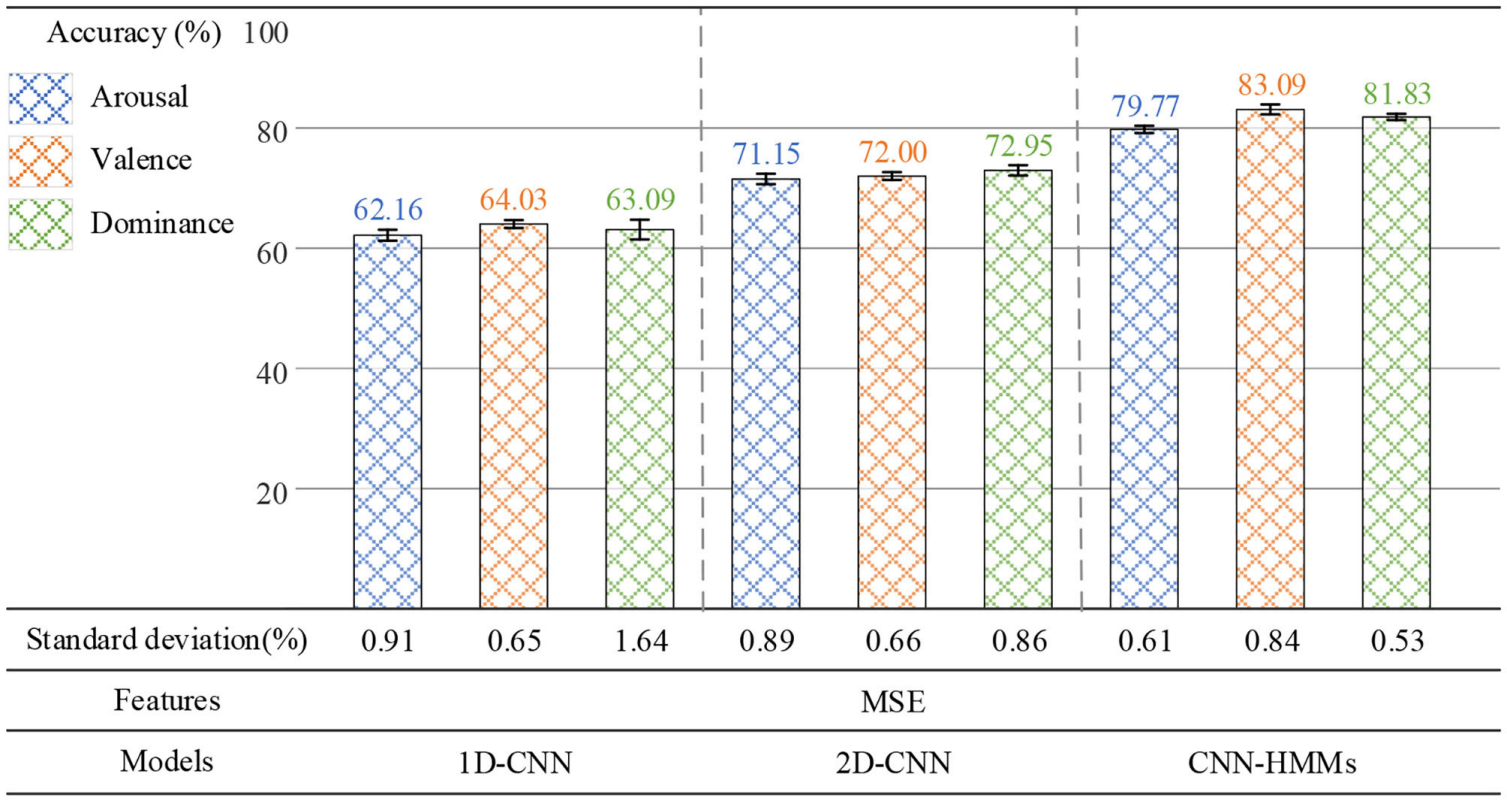
Results of the emotion recognition based on the 1D-CNN, 2D-CNN, and CNN-HMMs.

In the second group of experiments, the parameter settings of the 1D-CNN, 2D-CNN, and CNN-HMM are shown in Table 3, where Cov is the convolution layer and Fc is the fully connected layer. MSE was used as the EEG emotion feature. At the same time, the 1D-CNN, 2D-CNN, and CNN-HMMs were used for emotion recognition. As shown in Figure 11, the 1D-CNN achieved average recognition accuracy rates of 62.16% on arousal, 64.03% on valence, and 63.09% on dominance. The 2D-CNN achieved 71.15, 72.00, and 72.95%, while the CNN-HMMs achieved an optimal accuracy of 79.77, 83.09, and 81.83%. So, the deep hybrid network CNN-HMMs achieved a better emotion recognition performance than the 2D-CNN and 1D-CNN, which indicated that the proposed model could obtain the time information of EEG more effectively. The emotional recognition performance of the 1D-CNN was lower than that of the CNN-HMMs and 2D-CNN, and it indicated that the CNN-HMMs and 2D-CNN could obtain more spatial information from the 3D feature matrix which we constructed.

**Table 3:** Parameter settings of the 1D-CNN, 2D-CNN, and CNN-HMM.

**Models**	**Convolution kernel size**				**Neurons**		**Classifiers**
	**Cov1**	**Cov2**	**Cov3**	**Cov4**	**Fc1**	**Fc2**	
1D-CNN	1 × 8	1 × 4	1 × 4	1 × 2	1024	——	Softmax
2D-CNN	4 × 4	4 × 4	4 × 4	2 × 2	1024	——	Softmax
CNN-HMMs	4 × 4	4 × 4	4 × 4	2 × 2	1024	512	HMMs

### EEG Channel Activation

**Figure 12:**
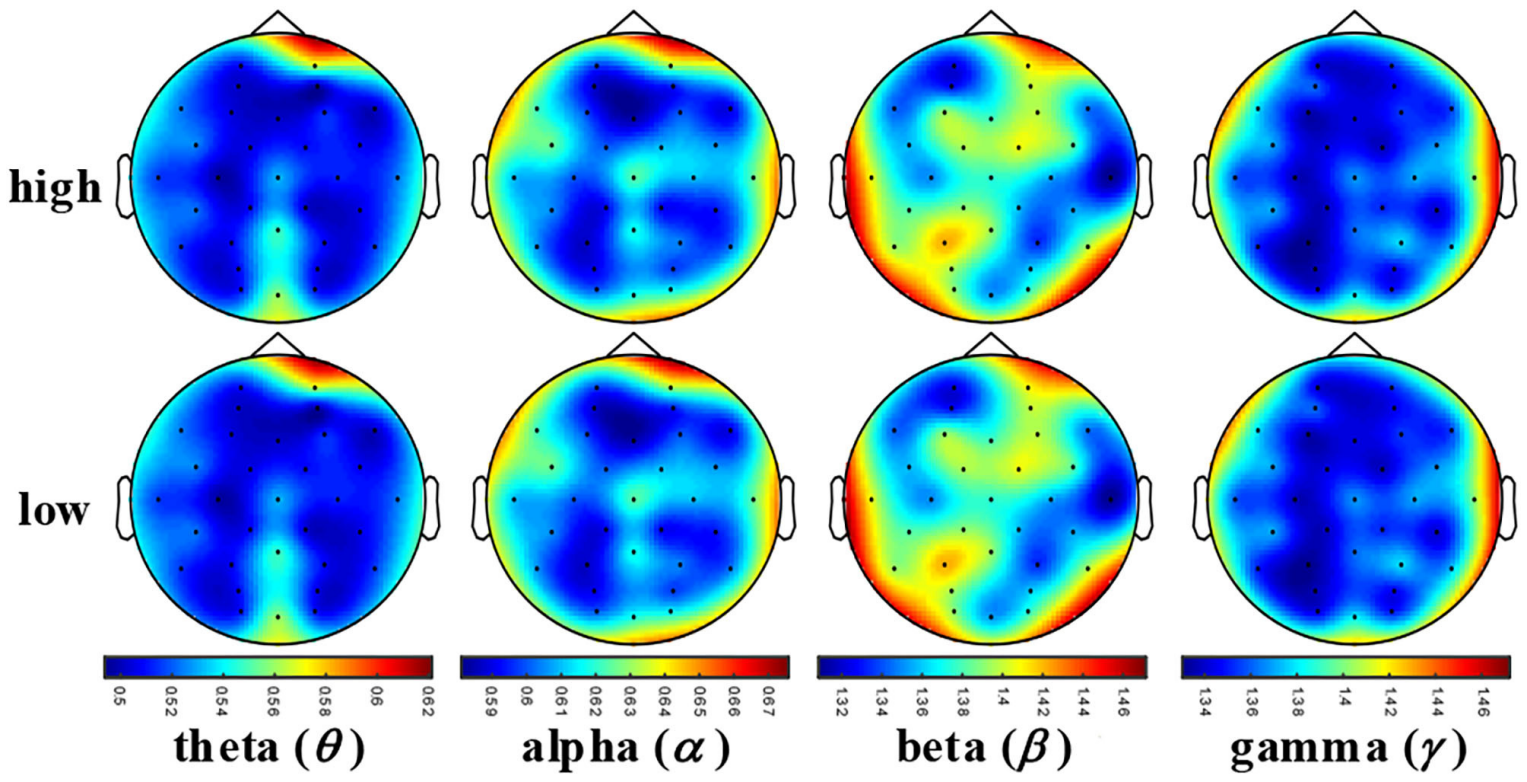
Scalp MSE distribution of two emotion states under different frequency bands.

In order to reveal the reason for the poor performance of emotion recognition and the EEG channels related to the emotional state, Figure 12 presents the averaged MSE distribution from all subjects, where each frequency band holds two activation topologies.

We found that the FP2 channel of the right frontal lobe, the O2 channel of the occipital lobe, the T7 channel of the left temporal lobe, and the T8 channel of the right temporal lobe had significant activation at the MSE distribution, indicating that these electrodes and brain regions are important for EEG emotion and are consistent with the results found in a previous study (Li et al., 2019b). We also observed that the same frequency band-related activation distributions for different emotional states are of a similar channel activation, which was the reason for the low performance of emotional recognition.

### Comparison and Analysis

We used deep hybrid network CNN-HMMs for emotional recognition based on EEG access and achieved the emotional recognition on arousal, valence, and dominance of the DEAP dataset. A 10-fold cross-validation technique was used to validate our emotion recognition results. At the same time, the proposed method was compared with existing methods.

**Figure 13:**
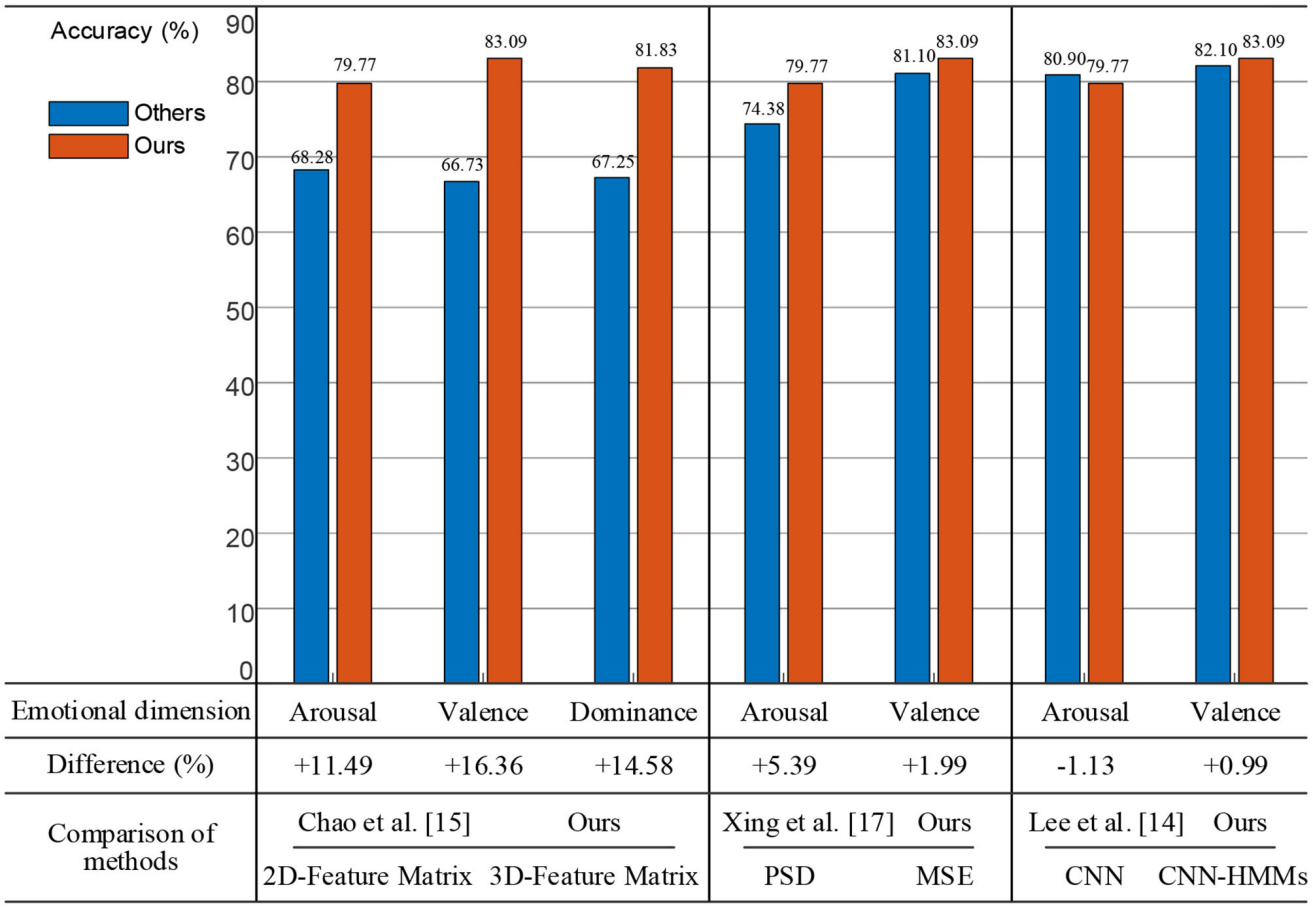
Comparison and analysis with existing methods.

Firstly, we constructed a 3D spatial feature matrix using four frequency band (θ, α, β, and γ) features of EEG and removed the baseline signals from the MSE features of the trail signals. As shown in Figure 13, a two-dimensional planar feature matrix was constructed by combining features of the four frequency bands (Chao et al., 2019). The experimental results showed that the proposed method achieved accuracy rates of 11.49, 16.36, and 14.58%, which were higher than theirs on arousal, valence, and dominance, respectively. Therefore, the 3D feature matrix could extract more useful EEG spatial information.

Secondly, we used the MSE method to perform nonlinear dynamics analysis of the EEG signals. As shown in Figure 13, power spectral density (PSD) was used to perform time and frequency domain analysis of the EEG signals (Xing et al., 2019). The experimental results showed that the proposed method was 5.39% on arousal and 1.99% on valence higher than theirs. Therefore, the MSE nonlinear dynamic method was more effective for EEG analysis.

Thirdly, on the basis of the CNN, we deeply fused the HMM model which had time series modeling capabilities. And the deep hybrid network CNN-HMMs were used for emotion recognition. As shown in Figure 13, a CNN was used to conduct the emotional analysis of the EEG and PPG signals (Lee et al., 2020). The experimental results showed that the proposed method was 0.99% higher on valence and 1.13% lower on arousal, which indicated that the combination of EEG and PPG signals was more effective for emotion recognition, but other physiological signal access would increase the complexity of actual emotion recognition.

Therefore, the proposed method could achieve the highest emotional recognition accuracy on valence and dominance, which was 83.09 and 81.83%, respectively. It was verified that the effectiveness of EEG access was best for the proposed emotion recognition method. A comparison of the related methods are shown in Table 4. Our method still has some limitations. On one hand, the proposed model requires 35.45 s when testing a group of EEG signals, which is not sustainable for hardware implementation. On the other hand, the accuracy of the emotion recognition obtained cannot meet the actual needs, so we will consider using an attention mechanism (Li et al., 2020), generative adversarial network (GAN) (Li et al., 2019a), or other advanced models for experiments.

**Table 4:** Results comparison of emotion recognition based on EEG access among similar studies.

**Studies**	**Models**	**Features**	**Evaluation methods**	**Accuracy (%)**		
				**Arousal**	**Valence**	**Dominance**
Chen et al. (2015)	HMM	Fusion feature	5-fold cross-validation	73.00	75.63	——
Li et al. (2016)	CNN-LSTM (CRNN)	Wavelet energy	5-fold cross-validation	74.12	72.06	——
Zhuang et al. (2017)	SVM	Intrinsic mode functions	leave-one-trail-out validation	69.10	71.99	——
Mert and Akan (2016)	ANN	MEMD-based features	leave-one-trail-out validation	75.00	72.87	——
Kwon et al. (2018)	2D-CNN	EEG spectrograms	10-fold cross-validation	78.12	81.25	——
Chao et al. (2019)	CapsNet	Multiband feature matrix	10-fold cross-validation	68.28	66.73	67.25
Xing et al. (2019)	LSTM	Frequency band power	10-fold cross-validation	74.38	81.10	——
Lee et al. (2020)	CNN	Fusion feature	5-fold cross-validation	80.90	82.10	——
Our proposed method	CNN-HMMs	Multiscale sample entropy	10-fold cross-validation	**79.77** **±** **0.61**	**83.09** **±** **0.84**	**81.83** **±** **0.53**

*Bold values indicate accuracy ± standard deviation (%)*.

## Conclusion

A method of emotion recognition based on EEG access was proposed by us in this paper. A 3D feature matrix, which was conducted by the multi-band MSE features of different EEG channels, could be extracted the EEG spatial information effectively. And a deep hybrid network CNN-HMMs, which was composed of a CNN and multiple HMMs, could be used to model the time series and perform emotion recognition. The proposed method was applied to the DEAP dataset for emotion recognition experiments and compared with the existing relevant studies, and it could achieve the highest average accuracy on valence and dominance. So, the proposed method could not only extract EEG features effectively, but could also improve the rate of emotion recognition.

In our future work, we will focus on reducing the recognition time and improving the recognition rate. Firstly, we will further study the correlation between the different electrode channels of EEG. In addition, we will utilize the method of rearranging channels to reduce EEG channels and select the optimum channels. Secondly, we will consider using a lightweight model. While the network parameters are reduced, there is no loss of network performance. In actual application, it is a competent choice for hardware implementation. In the meantime, we will also take into account the advanced deep learning models which will be used for improving the recognition rate.

## Data Availability Statement

Publicly available datasets were analyzed in this study. This data can be found here: http://www.eecs.qmul.ac.uk/mmv/datasets/deap/.

## Author Contributions

QZ and YZ designed this project. YZ and DC carried out most of the experiments and data analysis. LX and HZ revised the manuscript. All authors analyzed the results and presented the discussion and conclusion. All authors contributed to the article and approved the submitted version.

## Conflict of Interest

The authors declare that the research was conducted in the absence of any commercial or financial relationships that could be construed as a potential conflict of interest.
